# Proximal aortic stiffness modifies the relationship between heart rate and backward wave and hence central arterial pulse pressure

**DOI:** 10.3389/fcvm.2022.971141

**Published:** 2022-10-19

**Authors:** Nonhlanhla Mthembu, Vernice R. Peterson, Gavin R. Norton, Eitzaz Sadiq, Andrea Kolkenbeck-Ruh, Ravi Naran, Suraj M. Yusuf, Grace Tade, Hamza Bello, Adamu Bamaiyi, Carlos D. Libhaber, Patrick Dessein, Ferande Peters, Taalib Monareng, Talib Abdool-Carrim, Ismail Cassimjee, Pinhas Sareli, Girish Modi, Angela J. Woodiwiss

**Affiliations:** ^1^Cardiovascular Pathophysiology and Genomics Research Unit, School of Physiology, Faculty of Health Sciences, University of the Witwatersrand, Johannesburg, South Africa; ^2^School of Clinical Medicine, Faculty of Health Sciences, University of the Witwatersrand, Johannesburg, South Africa

**Keywords:** heart rate, aortic pressure, flow, forward waves, backward waves, age

## Abstract

**Aims:**

A lower heart rate (HR) increases central blood pressure through enhanced backward wave pressures (Pb). We aimed to determine whether these relationships are modified by increases in aortic stiffness.

**Methods:**

Using non-invasive central pressure, aortic velocity and diameter measurements in the outflow tract (echocardiography), we assessed the impact of aortic stiffness on relationships between HR and arterial wave morphology in 603 community participants < 60 years of age, 221 ≥ 60 years, and in 287 participants with arterial events [stroke and critical limb ischemia (CLI)].

**Results:**

As compared to community participants < 60 years, those ≥ 60 years or with events had increased multivariate adjusted proximal aortic characteristic impedance (Zc) and carotid femoral pulse wave velocity (PWV) (*p* < 0.05 to < 0.0001). Community participants ≥ 60 years and those with events also had a greater slope of the inverse relationship between HR and Pb (*p* < 0.001 for comparison). While in community participants < 60 years, no interaction between indexes of aortic stiffness and HR occurred, in those ≥ 60 years (*p* < 0.02) and in those with arterial events (*p* = 0.001), beyond aortic root diameter, an interaction between Zc and HR, but not between PWV and HR independently associated with Pb. This translated into stepwise increases in the slope of HR-Pb relationships at incremental tertiles of Zc. Although HR was inversely associated with the systemic reflection coefficient in community participants ≥ 60 years (*p* < 0.0001), adjustments for the reflection coefficient failed to modify HR-Pb relations.

**Conclusion:**

Beyond the impact on systemic wave reflection, increases in proximal aortic stiffness enhance the adverse effects of HR on Pb and hence central BP.

## Introduction

A lower heart rate (HR) is a well-established determinant of an increased central arterial (PPc), but not peripheral pulse pressure (PP) (1–3). A reduction in HR with β_1_ selective adrenergic receptor blockers increases PPc without modifying peripheral BP (4, 5). This effect is thought to in-part account for the limited ability of these agents to reduce the risk of cardiovascular events in hypertension when employed as first line-agents (6, 7). Although guidelines consequently recommend that β-blockers should not be used as first-line therapy for uncomplicated hypertension (8), little attention has been given to developing approaches to limit the adverse effects on PPc of any HR reducing agent, when required for use in cardiac conditions (9). In this regard, only more recent studies have identified the detailed mechanisms that explain HR relationships with central arterial pressure wave morphology (10). However, the factors that modify the impact of HR on PPc have not been determined. In this regard, detecting those most at risk for the adverse effects of HR on PPc may assist in planning therapeutic strategies that may minimize the impact of these effects. One possible factor that may modify the impact of HR on PPc is an increased aortic stiffness, a change that frequently accompanies the major risk factors for cardiac pathology.

Conventional thought is that a lower HR is associated with an increased PPc primarily through a prolonged filling period and hence ejection duration and stroke volume (SV) (11). The increased SV is thought to enhance forward traveling pressure waves (Pf) both through increases in peak aortic flow and ejection volume (reservoir pressure effect) (11). As increases in systemic flow may be advantageous in cardiac conditions, the potential deleterious impact of HR reduction on PPc in these conditions has been given little consideration. However, contemporary evidence shows that the relationship between HR and Pf is explained not by an increased SV or aortic flow, but because of an enhanced magnitude of re-reflected pressure waves generated by increases in reflected (backward traveling) pressure waves (Pb) (10). Importantly, in contrast to increases in SV or flow, which are potentially beneficial in cardiac conditions, increases in Pb create an impedance (resistance in a pulsatile system) to flow, which are likely to have adverse effects. The mechanisms that explain HR relationships with Pb include the inverse frequency dependency of the reflection coefficient and the harmonics of the pulse wave (which moves to a lower frequency at decreasing HR) (12). Thus, increases in aortic stiffness could produce two potential effects on the relationships between HR and Pb and hence PPc. Aortic stiffness increases aortic characteristic impedance to flow (Zc) and hence reduces the impedance mismatch between the aorta and more distal arterial vessels (1). As wave reflection occurs at points of impedance mismatch, an increased aortic stiffness could decrease the impact of HR on wave reflection and hence reduce the inverse relationship noted between HR and Pb. Alternatively, increases in aortic stiffness may also reduce the harmonic frequencies of the pulse wave (13) and increase the impact of a lower HR on Pb. Importantly, the effect of aortic stiffness on relationships between HR and central aortic pulse wave characteristics have not been determined *in vivo*. In the present study, we therefore aimed to compare relationships between HR and central arterial pulse wave morphology in those with either age-related increases in aortic stiffness (community sample ≥ 60 years of age) or arteriosclerotic-related arterial events that occur across the full adult age range in developing countries (14, 15) with a community sample < 60 years of age. We selected 60 years of age as the threshold (inflection point) at which age-related increases in aortic stiffness begin to markedly increase at a population level. This is indeed the age at which HR effects on PPc begin to produce clinically significant effects (10).

## Materials and methods

### Study groups

The present study was conducted according to the principles outlined in the Helsinki declaration. The Committee for Research on Human Subjects of the University of the Witwatersrand approved the protocols (approval numbers: M11-08-29, M14-04-29, M19-06-88, M16-04-11, M21-111-55, M02-04-72, M07-04-69, M12-04-108, M17-04-01, and M22-03-93). Participants gave informed, written consent. The present study design has previously been described (14–19). To obtain community participants either < or ≥ 60 years of age, nuclear families of black African descent (Nguni and Sotho chiefdoms) with siblings older than 16 years of age were randomly recruited (population census figures of 2001) from the South West Township (SOWETO) of Johannesburg, South Africa. In the present sub-study 824 participants had high quality aortic velocity measurements in the outflow tract. To obtain participants with arterial events, 287 consecutive black South Africans with stroke (*n* = 109) who did not have atrial fibrillation at the time of assessment or critical limb ischemia (CLI) (*n* = 178) with high quality aortic velocity assessments in the outflow tract were recruited from the Charlotte Maxeke Johannesburg Academic Hospital, South Africa. The presence of CLI or stroke was identified as described (14, 15). In this regard, black African patients attending the Charlotte Maxeke-Johannesburg Academic Hospital are of the same socioeconomic class as those living in the SOWETO community.

### Clinical and demographic information

A questionnaire was administered to obtain demographic and clinical data as described (19). Clinical information was also extracted from the hospital records and confirmed by the attending physician (14, 15). Clinical data included the presence of risk factors and the therapy thereof. Height and weight were measured using standard approaches and participants were considered to be obese if their body mass index (BMI) was ≥ 30 kg/m^2^. Laboratory blood tests of renal function, liver function, blood glucose, hematological parameters, and percentage glycated hemoglobin (HbA1c) were performed. Diabetes mellitus (DM) was defined as the use of insulin or oral glucose lowering agents, a fasting plasma glucose concentration ≥ 7 mmol/l or an HbA1c value greater than 6.5%. High quality office brachial blood pressure (BP) measurements were obtained in the seated position and after 5 min of rest, by a trained nurse-technician using a standard mercury sphygmomanometer as previously described (19) and according to guidelines. The mean of 5 measurements obtained at least 30 s apart was taken as office BP. Hypertension was defined as a mean office BP ≥ 140 mm Hg systolic or ≥ 90 mm Hg diastolic BP or the use of antihypertensive medication.

### Central arterial hemodynamic assessments

Central arterial hemodynamics were determined from central arterial pressure recordings using pulse wave analysis and aortic velocity and diameter assessments obtained in the outflow tract as previously described (14–18). After participants had rested for 15 min in the supine position, arterial waveforms at the radial (dominant arm) pulse were recorded by applanation tonometry and SphygmoCor software. Central arterial waveforms were generated from peripheral waveforms using a validated generalized transfer function in SphygmoCor software. Immediately after peripheral and central arterial pressure waveforms were acquired, aortic velocity and diameter measurements were obtained by an experienced observer (AJW) in the left lateral decubitus position using an Acuson SC2000 Diagnostic ultrasound system (Siemens Medical Solutions, USA, Inc.). Velocity waveforms were obtained in the 5-chamber view. High quality velocity assessments were identified as those with a smooth velocity waveform with a dense leading (outer) edge and a clear maximum velocity. Aortic diameter measurements were obtained just proximal to the aortic leaflets in the long axis parasternal view. The largest diameter recorded in early systole was used to construct an aortic flow waveform.

### Central arterial waveforms

Central arterial waveforms were generated as previously described (14–18) based on prior studies (20–22). Taking care to avoid any overshoot of the image, the leading (outer) edge or the most dense, or brightest, portion of the spectral image of the velocity waveform was outlined using graphics software. Aortic velocity and cross-sectional area were employed to construct a flow (Q) waveform. Characteristic impedance (Zc) was determined in the time domain using approaches previously described (20, 21) and validated against invasive pressure measurements (22). Using Zc values and flow and pressure waveforms, wave separation analysis was performed and Pb determined from (aortic PP – QxZc)/2 and Pf from (aortic PP + QxZc)/2 (14–18). The impact of HR on Pb independent of Pf was identified from reflection magnitude (Pb/Pf).

### Additional hemodynamic calculations

Heart rate (HR) was determined from the length (period, PD) of an averaged peripheral waveform captured over a 10 s period, using the formula: HR = 1,000/PDx60. The systemic (global) reflection coefficient was determined as (1 − Zc/SVR)/(1 + Zc/SVR) (12, 23), where SVR is systemic vascular resistance (or resistance vessel impedance, Zr) calculated from (mean arterial pressure-right atrial pressure)/cardiac output assuming right atrial pressure = 0 mm Hg. Carotid-femoral pulse wave velocity (PWV) was determined using standard approaches from SphygmoCor software (14, 15).

### Data analysis

For database management and statistical analysis, SAS software, version 9.4 (SAS Institute Inc., Cary, NC) was employed. Continuous variables are expressed as mean (SD or SEM). Dichotomous variables are expressed as percentages. For graphical representation of variables at different HR values, multiple variable adjusted data are shown across septiles of HR. Multiple linear regression analysis was performed to determine the independent relations between HR and hemodynamic variables. In regression analysis, adjustments were for age, sex, regular alcohol intake, regular tobacco intake, BMI, DM, mean arterial pressure (MAP) and the use of antihypertensive treatment. Probability values < 0.05 were considered to be significant. As age may affect the impact of aortic stiffness on HR-Pb relationships, sensitivity analysis was performed in those with arterial events < 60 years of age.

## Results

### Participant characteristics

The participant characteristics are given in Table 1. Arterial events occurred over an age range from 18 to 92 years. Participants with arterial events and community participants ≥ 60 years of age had a greater prevalence of several major risk factors including diabetes mellitus and hypertension, and regular smoking. Moreover, HDL cholesterol concentrations were lower in patients with vascular events. Although LDL cholesterol concentrations were also lower in cases, 86.6% of these patients were receiving lipid-lowering therapy at the time of obtaining fasting blood samples.

**TABLE 1:** Participant characteristics.

	Community sample		
	<60 years	≥60 years	Arterial events
Sample size (*n* =)	603	221	287
Strokes/CLI (%)	−	−	38/62
% Women	68.2	69.2	39.4**
Age (years)	37.9 ± 12.9	69.3 ± 7.1**	54.5 ± 14.7**
% regular smoking	16.4	10.9	38.5**
% regular alcohol	21.9	14.0	55.3**
% Hypertensive	35.2	81.0**	82.5**
% treated for hypertension	16.3	41.2**	59.1**
% Diabetes mellitus	7.5	30.0**	31.9**
HDL cholesterol (mmol/l)	1.42 ± 0.36	1.35 ± 0.29	1.09 ± 0.44**
LDL cholesterol (mmol/l)	2.59 ± 0.78	3.08 ± 0.77**	2.28 ± 0.92**
SBP/DBP (mm Hg)	123 ± 19/82 ± 12	142 ± 23**/85 ± 12*	127 ± 24*/80 ± 13
Heart rate (beats/min)	67 ± 12	68 ± 13	84 ± 17**

CLI, critical limb ischemia; HDL, high density lipoprotein cholesterol; LDL, low density lipoprotein cholesterol; SBP, systolic blood pressure; DBP, diastolic blood pressure. *p < 0.001, **p < 0.0001 vs. community sample < 60 years.

### Differences in central aortic function between groups

With adjustments for age, sex, and associated risk factors, in both community participants ≥ 60 years of age and in those with arterial events, an increase in carotid-femoral PWV and proximal aortic characteristic impedance (Zc) was noted as compared to community participants < 60 years of age (Table 2). Differences in Zc were attributed to variations in aortic stiffness as these differences were retained with further adjustments for aortic root diameter (Table 2). In sensitivity analysis conducted in participants with arterial events < 60 years of age, both PWV (*p* < 0.0001) and Zc (*p* < 0.02) were also increased as compared with age-matched community participants. As compared to community participants < 60 years of age, community participants ≥ 60 years of age and those with arterial events had increases in multivariate adjusted Pf and peak pressures generated by the product of Zc and Q (P_*QxZc*_) (Table 2). With adjustments, community participants ≥ 60 years of age, but not those with arterial events showed a trend for increases in Pb and RM (Table 2). In sensitivity analysis conducted in participants with arterial events < 60 years of age, Pf and P_*QxZc*_ were also increased compared with age-matched community participants (*p* < 0.0001), while neither Pb nor RM showed differences (*p* = 0.14 and *p* = 0.73, respectively).

**TABLE 2:** Multivariate adjusted central arterial function.

	Community sample		
	<60 years	≥60 years	Arterial events
Sample size (*n* =)	603	221	287
Central pulse pressure (PPc) (mm Hg)	33.5 ± 0.6	39.1 ± 0.9***	40.5 ± 1.2***
Carotid-femoral PWV (m/s)	6.02 ± 0.13	7.72 ± 0.22**	7.79 ± 0.39**
Peak aortic flow (Q) (mls/s)	351 ± 10	395 ± 16*	370 ± 17
Characteristic impedance (Zc) (dyne.cm^–5^)	80.7 ± 2.2	91.1 ± 3.1*	92.9 ± 4.3*
Backward wave pressures (Pb) (mm Hg)	13.1 ± 0.3	14.3 ± 0.4*	12.9 ± 0.6
Forward wave pressures (Pf) (mm Hg)	26.0 ± 0.5	30.1 ± 0.7***	30.3 ± 0.9**
Systemic reflection coefficient	0.897 ± 0.003	0.881 ± 0.004**	0.877 ± 0.006*
Aortic root diameter (mm)	25.6 ± 0.2	25.3 ± 0.3	25.2 ± 0.5
Reflection magnitude (RM = Pb/Pf)	0.48 ± 0.01	0.50 ± 0.01*	0.47 ± 0.02

P*_QxZc_*, Peak pressures generated by the product of flow (Q) and characteristic impedance (Zc). All data are adjusted for age, sex, MAP, BMI, heart rate, regular smoking, regular alcohol, DM, and antihypertensive therapy and comparisons of Zc are with further adjustments for aortic root diameter.*p < 0.05, **p < 0.005, ***p < 0.0001 vs. community sample < 60 years of age.

### Relationships between heart rate and reflected wave pressures and central arterial pulse pressure

**FIGURE 1:**
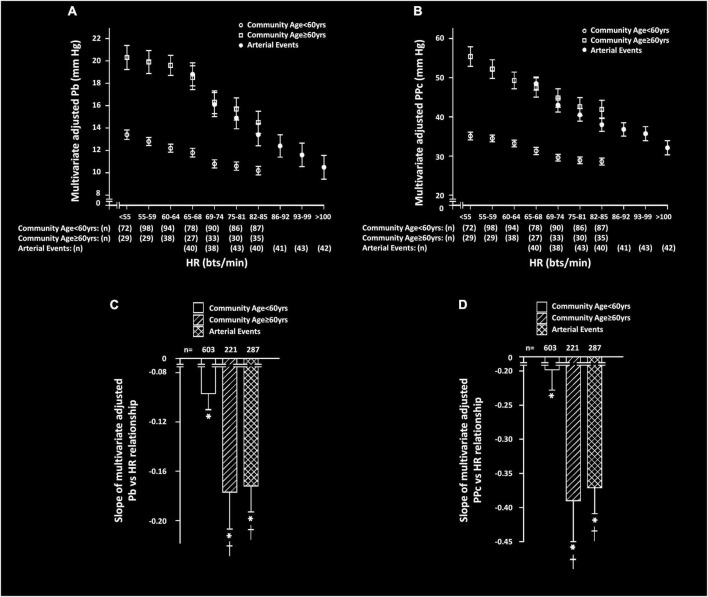
Multivariate adjusted backward wave pressures (Pb) or central arterial pulse pressure (PPc) across septiles of heart rate (HR) **(A,B)** and slopes of HR-Pb relationships **(C,D)** in participants from a community sample either younger (community < 60 years) or older (community ≥ 60 years) than 60 years or in those with arterial events (stroke or critical limb ischemia). Panels C and D show comparison of the multivariate adjusted slope (β-coefficient) of the relationships. All data are adjusted for age, sex, MAP, BMI, regular smoking, regular alcohol, DM, and antihypertensive therapy. *p < 0.0001 for relationships. ^†^*p* < 0.01 vs. slopes of relationships in community participants < 60 years.

Independent of confounders, HR was strongly and inversely associated with both Pb and PPc in both community participants and in those with arterial events (Figure 1 and Table 3). However, the slope of the regression relationship (β-coefficient) between HR and both Pb and PPc was increased in community participants ≥ 60 years of age and in those with events as compared to community participants < 60 years of age (Figure 1). In sensitivity analysis conducted in those with events < 60 years of age, the slope of the relationship between HR and Pb or PPc was similarly greater than in community participants < 60 years of age (β-coefficients ± SEM, HR vs. Pb; community sample = −0.097 ± 0.013, arterial events = −0.169 ± 0.022, *p* < 0.01 for comparison, HR vs. PPc; community sample = −0.198 ± 0.030, arterial events = −0.326 ± 0.040, *p* < 0.05 for comparison).

**TABLE 3:** Multivariate adjusted relationships (partial r) between heart rate (HR) and central arterial function.

	Community sample < 60 years			Community sample ≥ 60 years			Arterial events		
	Partial r (95% CI)		*P*-value	Partial r (95% CI)		*P*-value	Partial r (95% CI)		*P*-value
Sample size (*n* =)	603			221			287		
**Heart rate vs.**						
Central pulse pressure (PPc)	−0.256 (−0.330 to −0.179)		<0.0001	−0.359 (−0.471 to −0.234)		<0.0001	−0.463 (−0.550 to −0.365)		<0.0001
Peak aortic flow (Q)	0.052 (−0.029 to 0.132)		=0.21	−0.049 (−0.186 to 0.090)		=0.49	−0.055 (−0.209 to 0.102)		=0.49
Backward wave pressures (Pb)	−0.280 (−0.353 to −0.203)		<0.0001	−0.349 (−0.463 to −0.222)		<0.0001	−0.382 (−0.480 to −0.272)		<0.0001
Forward wave pressures (Pf)	−0.099 (−0.178 to −0.019)		=0.015	−0.207 (−0.333 to −0.074)		=0.0025	−0.245 (−0.354 to −0.129)		<0.0001
Systemic reflection coefficient	−0.134 (−0.212 to −0.054)		=0.0011	−0.248 (−0.370 to −0.116)		=0.0003	0.077 (−0.042 to 0.193)		=0.202
Reflection magnitude (RM)	−0.338 (−0.407 to −0.264)		<0.0001	−0.327 (−0.442 to −0.200)		<0.0001	−0.303 (−0.406 to −0.192)		<0.0001

P_QxZc_, Peak pressures generated by the product of flow (Q) and characteristic impedance (Zc). All data are adjusted for age, sex, MAP, BMI, regular smoking, regular alcohol, DM, and antihypertensive therapy.

### Impact of aortic stiffness on heart rate relationships with reflected wave pressures

**FIGURE 2:**
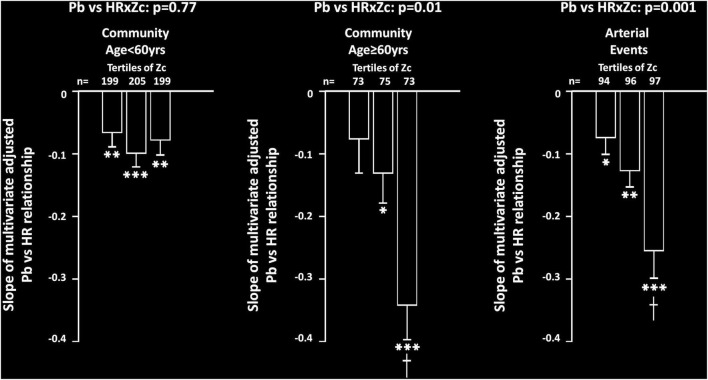
Impact of proximal aortic characteristic impedance (Zc) on multivariate adjusted slopes (β-coefficient) of relationships between heart rate (HR) and backward wave pressures (Pb) in participants from a community sample either younger (community < 60 years) or older (community ≥ 60 years) than 60 years or in those with arterial events (stroke or critical limb ischemia). Relationships are shown across tertiles of Zc in each group. All data are adjusted for age, sex, MAP, BMI, regular smoking, regular alcohol, DM, and antihypertensive therapy. *p < 0.02, ^**^p < 0.005, ^***^p < 0.0001 for relationships. ^†^*p* < 0.01 vs. slopes of relationships in first and second tertiles of Zc.

Beyond the individual terms and additional confounders, an interaction between Zc and HR was independently associated with Pb in both community participants ≥ 60 years of age (*p* < 0.02) and in participants with arterial events (*p* = 0.001). However, no independent interaction was noted between Zc and HR as a determinant of Pb in community participants < 60 years of age (*p* = 0.77). These interactions translated into a stepwise increase in the slope of the relationships between HR and Pb in community participants ≥ 60 years of age and in those with arterial events, while no increase in the relationship was noted in community participants < 60 years of age (Figure 2). In sensitivity analysis conducted in participants with arterial events < 60 years of age, an independent interaction between HR and Zc was similarly independently associated with Pb (*p* = 0.02). In contrast to the interactions between Zc and HR, in neither community participants ≥ 60 years of age (*p* = 0.12), nor in participants with arterial events (*p* = 0.24) was an interaction between HR and carotid-femoral PWV independently associated with Pb.

### Effect of the systemic reflection coefficient on heart rate-pressures relations

**FIGURE 3:**
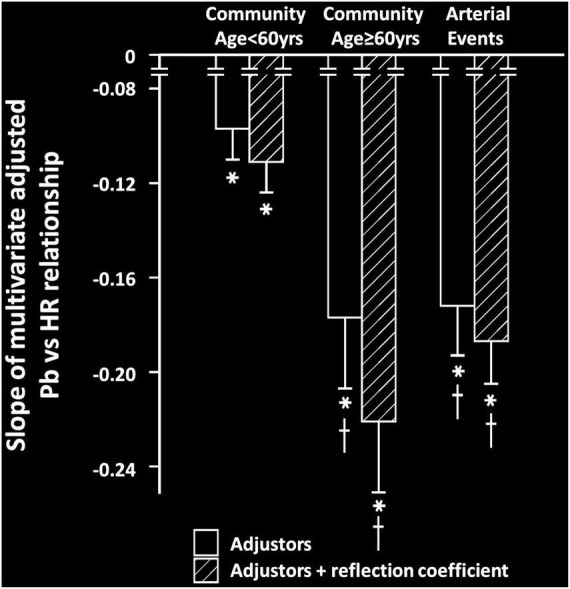
Impact of adjustments for the systemic reflection coefficient on the multivariate adjusted slopes (β-coefficient) of relationships between heart rate (HR) and backward wave pressures (Pb) in participants from a community sample either younger (community < 60 years) or older (community ≥ 60 years) than 60 years or in those with arterial events (stroke or critical limb ischemia). All data are adjusted for age, sex, MAP, BMI, regular smoking, regular alcohol, DM, and antihypertensive therapy. *p < 0.0001 for relationships. ^†^*p* < 0.01 vs. slopes of relationships in community participants < 60 years. No differences between relationships were noted before and after adjustments for the systemic reflection coefficient.

HR was strongly and independently associated with a decrease in the systemic reflection coefficient in community participants, but not in participants with arterial events (Table 3). Importantly, relationships between HR and Pb were unaffected by adjustments for the systemic reflection coefficient in either community participants < or ≥ 60 years of age or in participants with arterial events (Figure 3).

## Discussion

In the present study we assessed the impact of aortic stiffness on the relationships between a lower HR and increased central arterial backward wave pressures (Pb) and hence pulse pressure (PPc). As compared to community participants < 60 years of age, we noted an increased slope of these relationships in both community participants ≥ 60 years of age with an increased aortic stiffness and in those with arterial events (stroke and CLI) also with an increased aortic stiffness. The increased slope of the HR-Pb relationships in the groups with an increased aortic stiffness was explained by an interaction between proximal aortic characteristic impedance (Zc) and HR, independent of aortic root diameter. In this regard, in these groups, but not in community participants < 60 years of age, HR-Pb relationships showed stepwise increases across increasing tertiles of Zc. In contrast, no interaction between HR and carotid-femoral PWV was noted. Thus, increases in stiffness in the proximal, but not distal portion of the aorta accounted for the enhanced HR-Pb relations in groups with an increased aortic stiffness. Although HR was independently associated with the systemic reflection coefficient in community participants, adjustments for the systemic reflection coefficient failed to modify HR-Pb relationships in any group.

Through their limited benefits on outcomes (6, 7) β_1_ selective adrenergic receptor blockers are not recommended for use as first line agents in uncomplicated hypertension. However, underlying cardiac disease is a compelling indication for their use and they may be used as second, or third line agents even without cardiac disease (8). A well-recognized mechanism that may explain the adverse effects of β_1_ selective adrenergic receptor blockers is the increases in central, but not peripheral PP that occur with decreases in HR (2–5). Little attention has nevertheless been given to these possible adverse effects (2–5) as traditional thought is that the primary mechanisms responsible for these changes is an increased systemic flow, which may have possible beneficial effects in cardiac disease. However, more recent evidence indicates that decreases in HR increase central PP through an impact on Pb and not flow (10). As Pb creates an impedance to flow, the benefits produced by decreases in HR in cardiac disease may be offset by adverse effects on the LV. Therefore, identifying those most at risk of the adverse effects of HR reducing agents may improve approaches to the use of these agents in cardiac disease. In this regard, the present study is the first to show that those with an increased aortic stiffness are most likely to develop deleterious effects of HR reducing agents on central arterial PP and that strategies to manage the effects of HR reduction on central PP are therefore required in these patients.

The present study suggests that increases in aortic stiffness primarily reduce the harmonic frequencies of the pulse wave and in so doing enhance the magnitude of the pulse wave at lower HR values (13). In this regard, *in silico* studies demonstrate that the mechanisms that explain HR relationships with Pb include the inverse frequency dependency of the reflection coefficient and the harmonics of the pulse wave (which moves to a lower frequency at a decreasing HR) (12). In this regard, the reflection factor is well described as having primarily an inverse relationship with the frequency of the pulse in the ascending aorta (24). A lower HR is associated with lower frequency pulses in the aorta (12). As aortic stiffness increases aortic impedance, it decreases the impedance mismatch between the aorta and more distal vessels, an effect that will decrease the reflection coefficient. Thus, aortic stiffness may decrease wave reflection and reduce the impact of HR on Pb. However, in the present study, although HR was inversely associated with the reflection coefficient in community participants, adjustments for the reflection coefficient failed to modify HR-Pb relations in any of the groups. Thus, the dominant effect of HR on Pb is likely to be determined by harmonic effects on the pulse wave.

An older age is strongly associated with increases in Pb (18). As the average age of older community participants was greater than community participants < 60 years of age, it may be argued that the greater HR-Pb relations in those with an increased aortic stiffness can be attributed to an age-related increase in Pb. However, multivariate adjusted Pb values in those with arterial events was no greater than community participants < 60 years of age and HR-Pb relations were markedly increased in those with events. Moreover, in sensitivity analysis conducted in those with arterial events < 60 years of age, a markedly greater slope of the HR-Pb relationship was similarly noted as compared to community participants < 60 years of age. Hence, it is unlikely that an age-related increase in Pb is the explanation for the greater HR-Pb relations in those groups with an increased aortic stiffness.

There are several clinical implications of the present study. First, the present study explains the markedly greater relations between HR and central PP in those over 60 years of age (10). In this regard, older individuals have a greater aortic stiffness. Importantly, previous work suggests that the use of HR reducing agents in those younger than 60 years of age produces little clinical impact on BP (10). However, the present study indicates that even in those younger than 60 years of age with a high aortic stiffness, HR reduction will produce important adverse effects on BP that will not be detected at the peripheral pulse. Thus, PWV should be determined in younger individuals with risk factors and if HR reducing agents are required, approaches to limit these effects should be employed. Although speculative, the possible approaches to limiting the adverse effects of HR on Pb should therefore be considered. In this regard, although there is no proven ability to attenuate aortic stiffness, Zc may be reduced by decreasing aortic distending pressures (MAP). This may be achieved through the use of a variety of antihypertensives and intense brachial BP reduction may be required to decrease Zc and hence HR effects on Pb in those with increases in aortic stiffness.

The present study has several limitations. First, the present study was cross-sectional in design and hence causality may not be inferred. Further studies with HR reducing agents or with artificial pacing are required to identify whether aortic stiffness enhances the impact of HR on central PP. Second, the present study was not conducted in those with cardiac disease requiring HR reducing agents. Further work is therefore also required in patients with coronary artery disease or heart failure with a reduced ejection fraction who require HR reduction.

## Conclusion

In conclusion, in the present study we show that increases in aortic stiffness noted to occur in either the elderly or in high risk patients at any age, enhance the adverse effect of a reduced HR on central arterial PP. This occurs as increments in proximal aortic stiffness (as indexed by characteristic impedance independent of aortic root diameter) augment the increase in backward wave pressures that occur at lower HR values. As HR relationships with backward wave pressures occurred largely independent of the systemic reflection coefficient, these adverse effects of aortic stiffness are explained by the impact of stiffness on the harmonics of the pulse wave. These data suggest that aortic stiffness should be determined before HR reducing agents are initiated and that approaches to oppose the adverse effects of HR on backward wave pressures require identification in future studies.

## Data availability statement

The raw data supporting the conclusions of this article will be made available by the authors, upon reasonable request.

## Ethics statement

The studies involving human participants were reviewed and approved by the Human Research Ethics Committee of the University of the Witwatersrand. The patients/participants provided their written informed consent to participate in this study.

## Author contributions

All authors listed have made a substantial, direct, and intellectual contribution to the work, and approved it for publication.

## Abbreviations

BMI: body mass index
BP: blood pressure
CLI: critical limb ischemia
DBP: diastolic blood pressure
DM: diabetes mellitus
HbA1c: glycated hemoglobin
HDL: cholesterol high density lipoprotein cholesterol
HR: heart rate
LDL: cholesterol low density lipoprotein cholesterol
LV: left ventricle
MAP: mean arterial pressure
Pb: backward traveling (reflected) pressure wave
Pf: forward traveling pressure wave
PP: pulse pressure
PPc: central arterial pulse pressure
P_*QxZc*_: peak pressure generated by product of flow and characteristic impedance
PWV: carotid femoral pulse wave velocity
Q: peak aortic flow
RM: reflection magnitude (Pb/Pf)
SBP: systolic blood pressure
SV: stroke volume
SVR: systemic vascular resistance
Zc: aortic characteristic impedance
Zr: resistance vessel impedance.
